# Phenylbutazone (Bute, PBZ, EPZ): one drug across two species

**DOI:** 10.1007/s40656-018-0191-4

**Published:** 2018-03-26

**Authors:** Michael Worboys, Elizabeth Toon

**Affiliations:** Centre for the History of Science, Technology and Medicine (CHSTM), University of Manchester, Simon Building, Manchester, M13 9PL UK

**Keywords:** Phenylbutazone, Bute, Cortisone, Cross-species, Equine sports, Veterinary medicine

## Abstract

In this article we explore the different trajectories of this one drug, phenylbutazone, across two species, humans and horses in the period 1950–2000. The essay begins by following the introduction of the drug into human medicine in the early 1950s. It promised to be a less costly alternative to cortisone, one of the “wonder drugs” of the era, in the treatment of rheumatic conditions. Both drugs appeared to offer symptomatic relief rather than a cure, and did so with the risk of side effects, which with phenylbutazone were potentially so severe that it was eventually banned from human use, for all but a few diseases, in the early 1980s. Phenylbutazone had been used with other animals for many years without the same issues, but in the 1980s its uses in veterinary medicine, especially in horses, came under increased scrutiny, but for quite different reasons. The focus was primarily the equity, economics, and ethics of competition in equine sports, with differences in cross-species biology and medicine playing a secondary role. The story of phenylbutazone, a single drug, shows how the different biologies and social roles of its human/animal subjects resulted in very different and changing uses. While the drug had a seemingly common impact on pain and inflammation, there were inter-species differences in the drug’s metabolism, the conditions treated, dosages, and, crucially, in intended clinical outcomes and perceptions of its benefits and risks.

## Introduction

In the winter of 2013, Europe was gripped by a meat adulteration scandal. Investigations, first in the Republic of Ireland and then across the Continent, revealed that certain food products sold as beef contained horsemeat; indeed some products proved to contain *only* horsemeat, rather than the beef or other types of meat listed on the label. In the UK, media and public interest in the scandal focused on consumers’ disgust of unwittingly consuming a type of meat shunned by the nation, and led to calls for improvement in food standard monitoring. However, some experts voiced another reason for alarm—the presence in horsemeat of phenylbutazone, often known as “bute” in veterinary medicine (Poulter and Rush 2013). Phenylbutazone had been introduced in the early 1950s in human medicine to treat arthritis and other inflammatory musculoskeletal disorders, and was soon widely used to treat animals, especially horses, suffering from pain and stiffness. Initially a popular drug for both humans and animals, by the mid-1980s it had been banned from human use in most Western countries due to safety concerns. But it continued to be used for treating musculoskeletal disorders in animals, especially horses, which explained why some commentators feared it might turn up in food products containing horsemeat of uncertain provenance. In the event, no phenylbutazone was found in meat products; even if it had been detected, experts estimated that levels of the drug would be so low as to pose no threat to human health (Science Media Centre 2013).

In this article we explore the different trajectories of this one drug, phenylbutazone, across two species—humans and horses—from the mid- to the late-twentieth century in the United Kingdom (UK) and United States (US). Despite receiving almost no attention from medical historians, phenylbutazone was an important, widely used drug that had long caused controversy, and not just in 2013 when its presence in one species (the horse) seemed to threaten the health of another (humans). In human medicine, as we show, phenylbutazone began as life as a potential “wonder drug”, was then redefined as an effective source of symptomatic relief in rheumatoid disorders, and then caused alarm as some users suffered severe side effects and even death. Some medical professionals and, increasingly, consumer advocates called for the drug to be withdrawn, but rheumatology specialists countered that it provided effective relief in cases where other drugs had failed, and that long experience allowed them to manage the drug’s risks in those patients. In many ways, then, phenylbutazone’s trajectory would bear many similarities to (and even criss-crossed) the familiar paths taken by other post-war “wonder drugs”. At first specialists debated and negotiated how best to employ phenylbutazone, claiming that they best knew how to balance a promising new drug’s potential for benefit to relieve inflammation with its risk of harm. As the power of regulatory agencies and consumer advocates grew, so too did worries about the relatively small but persistent risk posed by the former wonder drug. With more alternatives to phenylbutazone available, its continued availability seemed harder to justify, which made a ban possible.

But phenylbutazone’s mid-twentieth century trajectory as an animal drug, commonly referred to as “bute”, was quite different. First adopted in animal medicine in the 1950s, bute took its place alongside aspirin as one of the commonly used anti-inflammatory drugs in horses, a place it still holds in the twenty-first century. The only calls for a ban were in animals intended for human consumption. However, from the 1960s on, bute developed a high public and veterinary profile because of growing controversies over its use with in horseracing, show jumping, and three-day eventing. By relieving pain, the drug allowed injured horses to take part in competitive events. This potentially damaged the long-term health of the animal for short-term gain, and was also unfair to competitors who had chosen not to enter their injured horses. Treatment with the drug “out of competition” was mostly accepted, in part because the drug was cleared from the horse’s body quite rapidly, and certainly much faster than it was in humans. However, questions arose about the fairness of competitions where some horses would be on the drug and some not, and, if allowed, what would be an acceptable “in-competition” dosage. On this issue, as we show, there was a conflict between those seeking to protect the horse, mostly veterinarians and animal welfare lobbyists, and horse owners and their connections, who often appeared to be only interested in the short-term gains of prize money and prestige. The latter points to important differences between human and animal medicine, and even within animal medicine. In veterinary practice when the “patient” is a working animal, the economic costs and benefits of treatment are primary, with the management of suffering the means to an end, not necessarily an end in itself (Woods 2012; Rollin 2006). With pets and companion animals there was a different calculus and variations with the condition treated (Schlünder and Schlich 2009).

How can we account for the different paths taken by this one drug, phenylbutazone, in human and veterinary medicine? A simple answer would be species biology: phenylbutazone is, we now know, metabolised and cleared from the body at different rates in different animals. But thinking about phenylbutazone’s careers in human and veterinary medicine reveals a more complicated story—or rather, two stories, trajectories that occasionally intersected and overlapped, but often did not. What then can these tell us about the interlocking and yet separate histories of medical science, human and veterinary medical practice, and complex dynamics of use and regulation? Our discussion of the history of phenylbutazone in humans and horses reveals the contingent and complex interactions of different species physiologies, medical and veterinary practices, pharmaceutical interests, and the cultural contexts of the use of drugs. Despite these differences, we show that there was a common thread: the continuing importance of clinical experience in shaping medical and veterinary attitudes towards the drug’s toxicity, and in determining who should make decisions about the drug’s use.

## Phenylbutazone and human medicine

The search for a single drug which would cure arthritis has gone on for as long as the disease has been known. If one could believe everything one reads this would appear to have been successful many times, the curious thing being that each year brings some discovery of a new “cure”—“off with the old and on with the new.” (Copeman and Mason 1954, pp. 77–78)

The Swiss drugs firm Geigy first introduced phenylbutazone in 1949, presenting it to doctors as a new, effective anti-arthritic agent. As such, phenylbutazone was expected to fill a niche previously occupied by two drugs with a similar therapeutic profile. The first of these, antipyrine (trade name “Phenazone”) debuted in the last decades of the nineteenth century. Doctors recommended it as an antipyretic to patients with fever, but it was also popular, especially in the United States, as a general painkiller that was purchasable without a prescription. Once aspirin was introduced, the use of antipyrine declined but there remained a niche for a powerful painkiller which was filled by antipryrine, also known as aminopyrine, or by its trade name “Pyramidon” (MacTavish 2004, p. 27). Aminopyrine gained favour amongst interwar clinicians, as its analgesic and anti-inflammatory properties made it useful for treating both rheumatoid arthritis and rheumatic fever (Rodnan and Benedek 1970, p. 158). However, by the late 1930s several observers had connected the extended consumption of aminopyrine with agranulocytosis or granulocytopenia, a rapid and often dangerous decline in the number of white cells in the marrow and the blood (Dyke 1936; Report 1947). In 1949, Geigy tried to mitigate the troubling side effects of aminopyrine by introducing a new intravenous formulation the company called “Irgapyrin”, an equal mixture of aminopyrine and phenylbutazone, with the latter serving as a solvent. However, trials soon showed that phenylbutazone itself was actually an effective analgesic and anti-inflammatory, so in 1952 Geigy marketed it on its own under the trade name “Butazolidin” (Dudley Hart and Johnson 1952).

Phenylbutazone debuted in a mid-twentieth century West where new “wonder drugs” seemed to promise radical improvement in, and even elimination of, a number of old scourges. Historians have written most extensively about the excitement that surrounded antibiotics, but in the immediate post-war years cortisone also attracted significant medical, press, and public attention. When cortisone appeared in 1949, enthusiastic observers suggested it would work the same miracle for chronic inflammatory diseases that penicillin had for infectious diseases (Marks 1991; Cantor 1992; Hetenyi and Karsh 1997; Bud 2007, pp. 11–13). However, clinicians soon determined that cortisone only relieved pain and inflammation, providing many rheumatic patients with considerable symptomatic improvement, but did not remove the causes of the underlying disease process (Benedek 2011). In other words, cortisone sparked a therapeutic revolution in symptom management, not as a cure. The drug quickly gained a place—albeit a prominent one—in an already substantial therapeutic armoury that included other pharmacological approaches once thought revolutionary, such as gold treatment and physical therapies (Forestier 1934, 1953; Rodnan and Benedek 1970) Clinicians in the 1950s thus saw cortisone trace out a trajectory that other drugs for rheumatoid disorders, including phenylbutazone, would mimic: initial enthusiasm for an apparently powerful preparation was soon followed by reports of side-effects, many of them debilitating and some life-threatening, and then debate amongst clinicians as to how to balance their experience of a drug’s benefits for many with growing knowledge about its risks for some.

Of course, this phenomenon of enthusiasm followed by lowered expectations and then integration into a range of therapies was not new, nor was it unique to arthritis treatment (Ackerknecht 1974). In their study of chlorpromazine’s integration into 1950s asylum practice in the Netherlands, Pieters and Snelders (2005, pp. 394–395) note several such cycles of “optimism and disappointment” in the modern history of psychiatric drugs, and term these “Seige cycles” after the German psychiatrist who first described them in the 1910s.^1^ But it is not surprising that mid-century rheumatologists, as they were beginning to see and call themselves (Cantor 1991), would find themselves becoming especially familiar with raised hopes followed by dashed expectations. As with psychiatric disorders, it would turn out to be extremely difficult to determine what counted as therapeutic success or failure when managing rheumatological complaints: patients and their illnesses varied significantly, assessing symptomatic improvement in chronic, poorly understood disorders often seemed more subjective than objective, and the powerful drugs employed could have unexpected systemic effects that in some cases profoundly outweighed any benefit they offered.

What were rheumatologists to do? Leading British clinicians W. S. C. Copeman and R. M. Mason suggested a judicious, negotiated path, whereby new arthritis drugs would be administered in cases where “simpler” therapies were inadequate, and those advocating for such new drugs would have to steer “a middle way” that maximised benefit appropriately (Copeman and Mason 1954, pp. 83–85, 117). For guidance on that “middle way”, specialist clinician-researchers dealing with arthritis and other rheumatoid disorders relied largely upon their colleagues. As a wealth of scholarship on mid-century reforms of drug governance has shown, in the immediate post-war years government agencies in both the UK and the US put fundamental controls on labelling, importation, and distribution, but left those seeking to determine how to utilise “wonder drugs” looking to fellow clinician-researchers. As discussed most recently by Podolsky (2015), in the case of antibiotics, academic infectious disease experts inserted themselves as “therapeutic rationalists” into conversations about the possibilities and perils of new drugs, in counterbalance to clinicians and the pharmaceutical industry. In the case of arthritis drugs, as we shall see, elite clinician-researchers seemed to have played a more substantial role as arbiters of good prescribing, at least initially. At meetings, in journals, and through regular review publications, they discussed their understanding of the risks and benefits of new pharmaceuticals, as drawn from trials or institutional series, and expected that this experience would then trickle down to a broader medical readership. In the United States such regular reviews appeared in the *Journal of Chronic Diseases*^2^ and rheumatology publications, while in the UK findings were often reported at meetings sponsored by the Empire Rheumatism Council (ERC).

These sources reveal that even amidst a generation of “wonder drugs” that triggered cycles of enthusiasm followed by disappointment, phenylbutazone offered an especially knotty array of benefits and risks for its advocates and detractors to evaluate. Differences of opinion emerged as early as 1952, when elite rheumatologists first conferred about their early therapeutic evaluations of phenylbutazone. After initial positive reports in the summer of 1952, the ERC hosted the first extended discussion of phenylbutazone in London at the end of that year (Laurence 1952; Symposium 1953a, b). The meeting was opened by Otto Steinbrocker of New York’s Lenox Hill Hospital, who immediately offered a dampening assessment: while phenylbutazone was a powerful analgesic, he noted that “its antirheumatic effect, if any, was slight” (Symposium 1953a). Furthermore, over one-fifth of his patients on short-term phenylbutazone therapy and one-third of those using the drug long-term reported side-effects. Administering phenylbutazone “was fraught with hazards”, he concluded, warning his colleagues against what he termed “undue optimism” (Symposium 1953a, p. 1213).

By contrast, the British contingent at the symposium was cautiously optimistic. Sheffield’s G. R. Newnes agreed that toxic effects could be seen in at least a quarter of patients using phenylbutazone, and these ranged from gastrointestinal disorders and skin rashes to oedema and agranulocytosis, which even resulted in one death in his study. Nevertheless, he argued that “[i]n spite of the high incidence of toxicity … the use of Butazolidin in rheumatoid disease was justifiable and that it was particularly useful in the rehabilitation of the more long-standing cases” (Symposium 1953a, p. 1214). A follow-up meeting and the production of more data in June 1953 led to a cautionary editorial about phenylbutazone in the *Lancet* (Report 1953). This unsigned editorial noted that British and American doctors were using phenylbutazone successfully to control ankylosing spondylitis, osteoarthritis, gout, and the symptoms of rheumatoid arthritis, but also that the “high incidence of toxic side-effects” described by these doctors made it clear that “phenylbutazone can be dangerous.” The solution to this dilemma was a familiar one, though: “The physician, as so often happens, has to weigh advantage against risk” (Report 1953; Royal Society of Medicine 1953).

A November 1953 ERC symposium on phenylbutazone, described as an “interim stocktaking”, offered elite rheumatologists another chance to revisit the weighing of advantage and risk (Symposium 1953a, b). Attendees agreed on the drug’s value in relieving suffering, but offered markedly different opinions as to whether it had a specific anti-rheumatic action and how its toxicity could be managed. Animal tests, usually on a rabbit’s paw, pointed to a suppressive action on inflammation, but this benefit was hard to measure clinically in humans, especially as it varied among patients and across the many conditions for which it was used. (Neither this nor the previous symposium mentioned toxic effects in animals, though.) As with cortisone a few years previously, enthusiasm faded: phenylbutazone did not have a fundamentally curative action, and the relief of pain and stiffness ended when treatment was stopped. New York’s Otto Steinbrocker returned to add more side-effects to those previously reported, now including duodenal ulcers and aplastic anaemia (severe reduction of all blood cells). The meeting’s summary report concluded that under supervision such side effects were manageable. Those supervising treatment just needed to remember that phenylbutazone persisted for a long time in the human body and that if the drug seemed to work for the patient in question, “the intake should be reduced as rapidly as possible to the minimal effective level” (Symposium 1953b, p. 1147). In a few short months, this “new drug [that] combats crippling diseases” (Laurence 1953) had become, for rheumatologists and other clinicians at least, a selectively useful but potentially toxic drug.

But this debate about dangers of phenylbutazone apparently did not curtail the drug’s market success, with this “highly controversial agent” being prescribed widely and extensively (Current Concepts 1957, p. 716). Throughout the late 1950s and the 1960s, the medical commentators who described phenylbutazone in journal articles, reviews, and textbooks recited a familiar litany of benefits and risks. Whether the discussion emphasised the benefit to the patient or the risk of adverse reaction, though, depended on the writer. Some assessments were remarkably positive, such as a 1957 review published in German and edited by H. K. von Rechenberg, who worked at the University Clinic in Basel and (perhaps not surprisingly) had close links with Geigy (Von Rechenberg 1957, 1961, 1962). Von Rechenberg acknowledged the side-effects associated with the drug (and continued to do so in later editions of his work), but argued that with careful attention to dosage and monitoring, “Butazolidin treatment does not carry excessive risks and can give excellent results” (Von Rechenberg 1962, p. 150).

Some clinician-researchers without links to Geigy, such as the leading British rheumatologist W. S. C. Copeman, agreed. In his history of the rheumatic diseases, Copeman (1964) described phenylbutazone as a prime example of the new generation of drugs that could “often give great relief” to rheumatoid arthritis sufferers (p. 170). Likewise, in Copeman’s influential textbook of rheumatic diseases, rheumatologist J. J. R. “Ian” Duthie (1964) of Edinburgh maintained that phenylbutazone had valuable uses despite its association with gastrointestinal problems. To mitigate these effects and make the most of the drug’s ability to enhance functional capacity, he suggested starting patients on a high dose of the drug, then rapidly cutting back to a much lower dose, since toxic reactions to phenylbutazone usually occurred in the first three months of treatment. Duthie noted that starting with a large dose would quickly make it clear whether the drug would work for the individual, and allow the clinician to stop it quickly if it did not. In other words, phenylbutazone was a tool for the careful physician who selected and supervised his patients, conducting periodic blood examinations and monitoring therapy and reactions (Duthie 1964, p. 221; see also Sperling 1969). This was borne out by a review done that same year by two of Duthie’s Edinburgh colleagues. They noted that reports of phenylbutazone’s haematological toxicity had declined since the middle 1950s, suggesting this was perhaps because clinicians had learned to take “greater care in the selection of patients to receive the drug” (McCarthy and Chalmers 1964, p. 1066). Other authorities, however, were less sanguine about phenylbutazone’s overall value, even in the hands of experts. For instance, the writer of a 1965 unsigned *BMJ* leader admitted the drug’s value for patients who failed to respond to other therapy, but then promptly qualified his evaluation with reference to the “disastrous” nature of some adverse reactions (Phenylbutazone 1965, p. 773).

Those occasional but disastrous reactions received more attention from two groups that were also gaining more authority in discussions about pharmaceutical benefit, risk, and safety: governmental regulatory agencies and consumers. As several historians have described (see for instance Abraham 1995, chap. 2 for an overview), in the US high-profile disasters such as the 1937 Elixir  Sulfanilamide episode triggered initial efforts before World War II to find a larger role for safety regulation. Then after the war, concerns about the high cost of new drugs drew legislators, policymakers, and consumers to consider whether central governments could, and should, play a more substantial role in adjudicating the value of these new drugs. In the early 1950s UK, the spiralling growth of the drugs bill in new National Health Service led officials to ask whether such expensive medications were truly worth their cost, although government bodies, mindful of the strength of the British pharmaceutical export market, decided to avoid cost regulations that would complicate their relations with industry. By the beginning of the 1960s, increasing concerns about drug safety would be propelled to the fore by the Thalidomide disaster. This resulted in the creation of the Committee on the Safety of Drugs (CSD), later the Committee on the Safety of Medicines (CSM), together with the system of “yellow card” adverse reaction reporting. Meanwhile, in the US, the Kefauver Committee hearings of the late 1950s crystallised and gave voice to consumers’ concerns about high drug costs and safety issues, such as those surrounding chloramphenicol (marketed by Parke-Davis as “Chloromycetin”). Although the regulations set out at first were weaker than those Kefauver advocated, the Thalidomide disaster meant that the pharmaceutical industry was more willing to cooperate in establishing a regulatory process that would countenance not only safety, but also efficacy, and thus a much increased role for the US Food and Drug Administration (FDA) in approving new drugs.

Available FDA correspondence around phenylbutazone in the 1950s and 1960s suggests that its staff did track discussion around the drug and potential dangers in this period. Their subject files are peppered with brief mentions of, and cross-references to, medical journal articles or conferences concerning blood dyscrasias and other problems associated with the drug, and even photocopies of clinical notes and death certificates stemming from some incidents. When undertaking enforcement actions, FDA staff emphasised the drug’s dangers. For instance, when a dentist’s widow proved to have pressured her son, also a dentist, to supply her with substantial amounts of both Butazolidin and Phenobarbital, enforcement personnel stressed the “very dangerous” nature of such drugs when used without a doctor’s close supervision.^3^ Likewise, in the middle 1950s, one staff member had pointed out to his colleagues that recent journal reports about toxic hepatitis attributable to Butazolidin might prove useful in encouraging district attorneys to cooperate in pursuing cases of illegal distribution of the drug.^4^ Meanwhile, members of the public wrote to the FDA—especially after the Thalidomide scandal broke—with concerns about phenylbutazone, usually asking whether its use was legal, safe, and/or appropriate to their own cases. Responses from FDA personnel to these consumers (or in some cases their angry relatives) noted the drug’s official status, and tended to bounce any questions about safety or appropriateness back to the patient’s own clinician. Manufacturers were required to supply full information to physicians, FDA staff assured those who wrote them, and then it was up to the physician to decide “whether the benefit the drug is expected to provide will reasonably offset the disadvantage of side effects it may possibly cause”.^5^ But such reassurance did not stop such officials from noting their suspicions about some consumers' concern; the same official, for instance, noted in a later case that a particular correspondent “obviously was attempting to find some point which would be of advantage in a malpractice suit”.^6^

Meanwhile, from the middle 1950s onward, pharmaceutical companies offered new drugs that could be alternatives to phenylbutazone, such as the new corticosteroids prednisone, methylprednisolone, triamcinolone, and dexamethasone, all of which were claimed to be both safer and more effective. These new corticosteroids attracted medical and public attention, and, buoyed by aggressive marketing campaigns, competed with each other as well as with the older therapies (aspirin and gold treatment) and with phenylbutazone. Nevertheless, Geigy’s phenylbutazone continued to enjoy an important place in the market, as clinicians on both sides of the Atlantic used it with patients suffering from rheumatoid arthritis, osteoarthritis, ankylosing spondylitis and gout. Even so, Geigy caught the same enthusiasm and sought to develop an alternative to phenylbutazone. Like its competitors, the company was anxious to capitalise on a growing market for chronic conditions and enthused by the high profit potential from patent-protected drugs, and all in all the major pharmaceutical companies screened hundreds of compounds seeking a less toxic, more effective alternative to available treatments for acute and chronic inflammatory conditions. Geigy’s alternative, oxyphenbutazone, appeared at the end of the 1950s. This drug, which was marketed as “Tanderil” (“Tandearil” in the US), was seen to be a promising anti-arthritic, but unlike its parent, not useful for gout or pain relief. The market leader in anti-arthritic drugs, Merck, found that its dominant position in steroid drugs was under threat on two fronts, from other new steroids developed by competitors and from the growth of tighter regulations on drug safety (Sarett 1990). In their search for alternatives with fewer adverse effects, Merck’s scientists created indomethacin (trade name “Indocin”), a new class of drug with sufficient anti-inflammatory effects to move from laboratory promise to clinical acceptance, after being trialled by selected clinicians in the US and UK. Merck released indomethacin in 1963, styling it not only as a new chemical entity, but as a member of its new class of non-steroidal anti-inflammatory drugs, or NSAIDs, a term invented by researchers at Merck’s laboratories in New Jersey and West Point, Pennsylvania (Dudley Hart and Boardman 1963; Shen et al. 1963). The phrase “non-steroidal” was intended to distance the drugs from corticosteroids, the risks of which had become more apparent by the early 1960s. Along with aspirin, phenylbutazone and oxyphenbutazone were then retrospectively designated NSAIDs. Indomethacin itself, meanwhile, was used by many clinicians for various inflammatory conditions, particularly as its side-effects, which included nausea, vomiting, diarrhoea, headaches, hypertension and cardiovascular problems, were said by many doctors to be less serious and easier to manage than the toxic effects of steroids and phenylbutazone (Benedek 2011). Indomethacin’s success would then in turn fuel the search for similar NSAIDs. Over the next several decades, some of these (such as benoxaprofen, see below) would be rejected due to safety concerns, while others would become mainstays of prescribing and self-medication, notably ibuprofen, naproxen, piroxicam and celecoxib (Abraham 1995; Brooks and Buchanan 1985).

Despite indomethacin’s introduction, phenylbutazone continued to have its proponents amongst both specialists and general practioners, even as they observed more problems related to this drug and its cousin oxyphenbutazone. For instance, in the 1970 edition of Copeman’s rheumatology textbook, Edinburgh consultant Ian Duthie’s revised chapter on rheumatoid arthritis included a longer, and more serious, list of side effects than the 1964 edition had. Alongside the now well-known gastrointestinal issues associated with phenylbutazone, Duthie (1970) pointed out that some patients experienced agranulocytosis, aplastic anaemia, stomach ulcers, or thrombocytopenia (reduction in platelets in blood). Still other patients reported “headache, giddiness, sore mouth, blurring of vision,” but Duthie noted that patients on a placebo noted the same effects with the same frequency (p. 304). He concluded, much as he had in 1964, that despite these side effects phenylbutazone still had value, at least in the hands of the experienced practitioner treating the patient with few other options. This was in marked contrast to his evaluation of indomethacin, which he argued had side effects so toxic that it probably had no place at all in the treatment of rheumatoid arthritis (p. 305). By comparison, clinicians had long experience using phenylbutazone for rheumatic conditions and gout—and it was also the cheapest NSAID, aside from aspirin. Such evaluations are a strong reminder that despite reform efforts by proponents of what was called “rational therapeutics”, prescribing choices in this period were still frequently governed, even amongst specialists, by individual and local clinical experience (Podolsky 2015).

What finally led to phenylbutazone’s decline as a human drug was a coalition between consumer advocates and their allies within the medical profession, and lawsuits. On both sides of the Atlantic, activists and groups claiming to represent health consumers drew media and legislative attention to questions about drug safety and drug cost, resulting in high profile journalistic coverage as well investigative hearings (Tomes 2016, pp. 240–248 and 265–267; Mold 2015, pp. 119 and 123–124; see also Podolsky 2015). Consumer groups like Social Audit in the UK and the Health Research Group in the US, often supported by sympathetic medical professionals, subjected phenylbutazone and oxyphenbutazone to significant public criticism. The blood disorders and ulcers attributable to these medications were worrying enough, but the fact that these drugs could, in rare but highly visible cases, cause rapid death from aplastic anaemia strengthened demands for an outright ban. A study by the UK Committee on the Safety of Medicines (CSM), published in 1977, found the relative risks to be low for patients given short courses, but pointed to concerns about the drug in the elderly and when given long-term (Inman 1977; Committee 1978).^7^ Soon after, the CSM reviewed the safety profiles of several NSAIDs. Benoxaprofen, marketed as Ofren in the UK and Oraflex in the US, was an early casualty (Abraham 1995, chap. 4). This drug had only been on the market for two years in the UK and had just come on to the market in the US when, in the summer of 1982, the UK government suspended sales of the drug in response to reports of patient deaths and other adverse effects. When the FDA announced that it also had attributed a number of deaths to benoxaprofen, the drug’s maker Lilly immediately removed it from the market.

The benoxaprofen episode ratcheted up consumer, media and medical concerns about phenylbutazone and oxyphenbutazone. After all, these drugs had long been known to cause problems, and they had also been far more widely and extensively used than the newer NSAIDs: by the early 1980s, an estimated 180 million patients worldwide had used these drugs in the three decades since phenylbutazone’s introduction (Veitch 1983). But medical critics and consumer advocates would no longer stand for clinicians being the ones to determine what level of risk was acceptable. In early spring 1983, the UK’s Channel 4 aired an episode of its new series on drug safety *Kill or Cure?* devoted to the hazards of phenylbutazone and oxyphenbutazone, and released an accompanying booklet on drug injury claiming that phenylbutazone was an “unnecessary drug” (Brown 1983; Shenton and Adams 1983, p. 17). Later that year, activist professionals and consumer organisations obtained a smoking gun that would bolster their efforts. In February 1983, a confidential internal Ciba-Geigy memo estimated that its Butazolidin and Tanderil had caused nearly 1200 deaths.^8^ This memo was leaked to an outside clinician, Swedish paediatrician Olle Hansson, who in turn passed it along to the press (Paul 1984). In the UK, the CSM had gathered its own data supporting this evaluation, estimating that probably some 1500 patients had died as a result of taking phenylbutazone (Veitch 1983). Reaction around the world was swift: Norway, for example, instituted an outright ban on the drugs. In the US, campaigner Sidney Wolfe of the health consumer group Public Citizen promptly petitioned the Department of Health and Human Services (DHHS) to have phenylbutazone and oxyphenbutazone banned there. DHHS Secretary Margaret Heckler declined to do so, but did call for a “comprehensive report” on these drugs (Silverman et al. 1992, pp. 16–17; Veitch 1983).

Matters came to a head on both sides of the Atlantic in the spring of 1984. In the US, the benoxaprofen episode and continuing investigation of phenylbutazone and oxyphenbutazone continued to raise suspicion about NSAID safety generally. This timing could not have been worse, as the FDA was at that moment considering whether to allow ibuprofen to be sold over the counter, at lower doses than prescription. Critics suggested that consumer pressure around these drugs, along with previous controversies about other NSAIDs (aired in a 1982 congressional investigation into Oraflex and Zomax), contributed to the FDA’s slow progress in approving NSAIDs (and drugs generally). One US pharmaceutical executive even suggested that the FDA had come down with “Oraflexia nervosa…a fear of approving non-steroidal anti-inflammatories” (Schiebla 1984, p. 22).

In the UK the licence for oxyphenbutazone was withdrawn in 1984 and the next year phenylbutazone was restricted to hospital treatment of ankylosing spondylitis (Loshak 1985). Nevertheless, some clinicians still wanted to use phenylbutazone, and Ciba-Geigy sought to respond to this demand. In February 1985, the company convened a meeting in London to debate the issues. Professor von Wartburg, the senior Ciba-Geigy representative, argued that:

Simply taking them off the market is too easy an answer to a very complex issue. It would deprive many people of drugs they currently use. It would contradict the findings of many regulatory authorities which have reviewed the drugs, on our recommendation in the light of growing contraindications. It would merely shift the risk because rival (but not necessarily superior) products would replace them (Loshak 1985, p. 410).

He added that “We are not a money-making machine or greedy Swiss gnomes without a conscience” (Loshak 1985, p. 410). In reply, Dr Andrew Herxheimer, editor of the *Drug and Therapeutics Bulletin* and contributor to the *Kill or Cure?* series, claimed that the two drugs had been obsolete for at least 20 years, seemingly referring to the arrivals of new NSAIDs, and said it was now time for both to disappear (Herxheimer et al. 1985). In the end, Ciba-Geigy sought a compromise. They recommended that phenylbutazone become a drug of second choice, used only when “other non-steroidal anti-inflammatory drugs, had been tried and found unsatisfactory” and be reserved for the treatment of ankylosing spondylitis (Loshak 1985; Report 1985, p. 882). Some UK rheumatologists were unhappy. They complained that they had lost another NSAID, as they had lost fenclofenac, benoxaprofen and feprazone, and that regulatory authorities were not only causing unnecessary suffering, but also telling rheumatologists that their “training and experience is inadequate to use the drugs of our specialty” (Struthers et al. 1984, p. 318). In the US, meanwhile, the DHHS declined to ban phenylbutazone and oxyphenbutazone, but Secretary Heckler did approve the FDA’s recommendation calling for restrictive labelling for the drugs.

The two compromise approaches taken in the US and UK—restrictive labelling or restriction to hospital patients—were relatively successful in limiting phenylbutazone’s usage in these markets. In April 1985, Ciba-Geigy decided to halt worldwide sales of its “Tanderil”, stating that while the limitations on “Butazolidin” had been observed, those on their preparation of the more dangerous oxyphenbutazone had not (Aronson 2009, p. 347). Butazolidin and other, newer NSAIDs, they suggested, could meet existing demand, especially if governed by stricter controls (as in the UK’s restriction to hospitals only) or if presented with additional warnings and guidance, as in the US. And although phenylbutazone continued to be widely available, with few controls, in many developing countries, its use—whether as Ciba-Geigy’s “Butazolidin” or in its generic forms—dwindled in the face of an increasingly successful consumer strategy: lawsuits (Mintz 1986). Soon, phenylbutazone was withdrawn from human medicine in the UK, US, and other Western countries, except for the treatment of ankylosing spondylitis (Hart and Huskisson 1984; Faich 1987). But phenylbutazone’s story did not end here, as it remained an important and widely used drug—for animals.

## Bute in animal medicine

Veterinarians in the early 1950s took up phenylbutazone, which they commonly called PBZ, as enthusiastically as their medical counterparts had. They used it to treat joint afflictions, particularly in horses where its anti-inflammatory action countered lameness, the main reason that prevented a horse’s ability to work or to be ridden in sport or recreation. Phenylbutazone was relatively cheap, came in many formulations, was easy to administer and experienced to be highly effective, with minimal, if any, side effects. The veterinary use of drugs was not regulated in the same way as in human medicine; indeed, their usage was often very different, as shown by the adoption of antibiotics to enhance growth in livestock and poultry, which remained uncontrolled in Britain until the early 1970s (Bud 2007, pp. 163–191). The animal pharmaceutical market differed to that for human medicines in four main ways: (i) bulk supplies were often needed to treat herds or groups, (ii) the drugs available were more limited and included classes rarely used in humans (e.g. more antihelminthic and other parasiticides), (iii) there were fewer prescription-only medicines, and (iv) the market included more nutritional and other supplements. When treating specific diseases, veterinarians often supplied the drugs they prescribed; however, farmers and stables that required bulk quantities relied on veterinary supply companies, such as Arnolds Veterinary Products, the Veterinary Drug Company and Willington’s Medicals.

The administration and assessment of veterinary drugs in this period remained empirical, with no drive for clinical trials as in human medicine. Veterinary practices were private, operating as small businesses where veterinarians worked as general practitioners as individuals or in group practices. Though many veterinarians developed specialist expertise and took referrals, they were more professionally isolated than general practitioners and consultants, who saw themselves as members of a national (no doubt aided by the creation of the National Health Service) and increasingly international profession. There was seemingly little time or inclination to publish on their work. This was evident with phenylbutazone, where the first article on the drug in the *Veterinary Record* appeared in 1967, many years after bute's adoption in veterinary practice. This article dealt with the death of a dog (Tandy and Thorpe 1967), and the following year the *British Veterinary Journal* published an article on the drug in cats (Carlisle et al. 1968). In the UK, the 1968 Medicines Act, brought in after the Thalidomide scandal and which required evidence of efficacy and safety, also covered veterinary drugs. There were many grey areas with veterinary medicine with drugs of longstanding and non-pharmaceutical remedies. Phenylbutazone was placed on the most restricted list of drugs only to be prescribed by a veterinary surgeon.

The use of phenylbutazone in horses also had a low profile until it was found in the urine of the winner of the 1968 Kentucky Derby (Hunt et al. 2014). Bute had become a standard treatment for racehorses suffering from stiff and painful leg joints; these were especially a problem among racehorses because their training involved a mix of long, daily gallops to build stamina and short, intensive work-outs to develop speed. Short-term administration of bute could allow a horse to take part in a particular race, while it could also be administered long-term to keep a top-performing horse in training. The horse Dancer’s Image was subsequently demoted to last place and the prize awarded to the second horse, a decision that was contested in the courts for several years and remains controversial to this day (Toby 2011). The use of drugs and other performance altering methods in horse racing is known to have had a long history, with interested parties seeking either to enhance or hinder a horse’s running, the racing term was “to stop” a horse. Nevertheless, there are few detailed accounts of the history of doping in horse racing. Histories of the sport have focussed on the racing itself—great horses, jockeys, trainers and owners, or its social history—from the “Sport of Kings” to the cultures of betting (Longrigg 1972; Vamplew 1976; Cassidy 2002; Huggins 2003; Rossdale et al. 2011). Sources for a history of doping would be problematic, as the activity was necessarily secretive and often criminal. Its practice was multi-faceted, with many ways and means to alter the performance of a horse, which were difficult to get right, even before the avoidance of controls added to the complexity (Clarke 1962; Higgins 2006). Some owners and trainers argued that boosting stimulants should be allowed as equivalent to the other main ways of boosting performance—breeding and training. However, “stopping” with depressants could in no circumstance be open as they affected fairness in racing and betting: either criminally “nobbling” a rival, or “stopping” one’s own horse in several races, to lengthen its odds to win heavily in a later race.

Concern about “doping” in horseracing came to the fore at the turn of the twentieth century, with controls that aimed to ensure fairness. The overarching powers of the British Jockey Club allowed them to ban “doping” from 1903 and introduce very severe punishments for trainers. The term “doping” was adopted from racing in the US; indeed, the practice was seen to be more common there and could involve electrical stimulation with a belt under the saddle as well as the administration of drugs (Atkinson 1900; Report 1900). Testing was only for alkaloids, such as morphine and cocaine, and the rule only covered the race day; drugs were allowed in training before a race and after in recovery. The American Jockey Club had similar ambitions for banning “dope”, but their authority was shared with state Racing Commissions whose rules varied and were unevenly enforced. Doping remained commonplace in the American racing and only began to come under scrutiny with the introduction of saliva testing in the early 1930s (Clarke 1962). However, these measures were undermined by the burgeoning production of new drugs after the Second World War, as the pharmaceutical industry fed both the hopes (and suspicions) of some trainers and owners, and race “fixers” that novel, undetectable compounds might give their horses an advantage, or provide new ways of “stopping”. Racing authorities instituted stricter rules and more effective testing regimes, but it took some time for these to come into force. In the UK major changes followed the report of a commission chaired by the Duke of Norfolk in 1961, following a high profile legal case involving the famous trainer Vincent O’Brien (Miller and Moss 1964; O’Brien and Herbert 2005, pp. 145–161.). One key principle remained: with only a few exceptions, no horse should be “medicated” on race day, which meant that trainers administering drugs to their animals needed to withdraw them at a suitable time before the race. The situation in the US, meanwhile, continued to be fractured, with changing and different rules in different states. For instance, in 1968, the year of Dancer’s Image’s demotion, many states allowed phenylbutazone and Kentucky itself lifted its ban in 1974. It was reported that, in 1986, 13 of the 16 horses running in the Kentucky Derby were being given phenylbutazone.

Phenylbutazone did not directly enhance performance; its value was in its anti-inflammatory and painkilling properties, which allowed a lame horse to continue training and competing. In the jurisdictions that only allowed bute in training, the crucial question was how long the drug remained active in the horse’s body. Users wanted to know how long before a race the drug needed to be withdrawn, while authorities wanted to be able to detect and monitor its use. This issue thus drove investigations into phenylbutazone’s pharmacokinetics in horses; knowing how the drug was metabolised and excreted, and being able to identify its residues and metabolites in blood and urine. A 1960 study, which was principally concerned with the drug in laboratory animals, had shown that phenylbutazone was comparatively rapidly metabolised in the horse (Table 1). In fact, bute’s half-life in the horse was twelve times shorter than in humans, who had by far the longest half-life of all the other animals tested, suggesting a problem for those who expected to use standard laboratory animals as models of human reactions (Burns et al. 1960; see also Burns 1968). Moreover, in horses the drug’s effects seemed to persist at very low plasma and tissue levels, perhaps even, experts mused, at levels that testing could not detect. These clearance properties meant that, even where it was banned, phenylbutazone could be used in horses quite close to the time of competition, a finding that, regulators worried, encouraged misuse and cheating.

**Table 1:** Species differences in the metabolism of phenylbutazone (Burns et al. 1960, p. 257.)

Species	Biological half life in hours
Man	72
Dog	6
Rabbit	3
Rat	6
Guinea pig	5
Horse	6

That there were species differences in the rates at which phenylbutazone was metabolised had been known since the early 1950s (Burns et al. 1953), but this knowledge seems to have had little impact amongst veterinarians. There was no citation of the Burns et al. (1960) paper in the veterinary literature before the early 1970s (see Bogan 1972; Moss and Haywood 1973), nor any published concerns about the drug’s safety (Dalton 1956). This dearth of literature on phenylbutazone’s benefits, if not its safety, is surprising given the drug’s popularity, especially in treating lameness in horses. It seems veterinarians in both the UK and US regarded bute, based on their clinical experience, as effective and safe, as well as easy to administer since it was conveniently available in both oral and injectable formulations. The contrast in its toxicity between humans and horses was ascribed to different half-lives. Yet this remained speculation as toxic effects often came from metabolites, of which there would have been a greater quantity in the horse due to the larger dosages (Anon 1979).

The 1968 demotion of Dancer’s Image led to new interest in phenylbutazone in the horse among US academic and equine veterinarians, the latter an emerging group within an increasing differentiated profession (Jones 2003). The *Equine Veterinary Journal* published a series of articles in 1972 on the issues around its use in all equine sports, but focused on the drug’s “misuse” rather than its potential toxicity (Dunn 1972; Hopes 1972; Moss 1972). A subsequent 1977 report argued that “PBZ does not change a horse’s innate ability to race, but by relieving inflammation it may enable the horse to race closer to maximum capabilities”, and further concluded that “Side effects due to phenylbutazone are unusual in horses, there being few in clinical experience or in horses in racing” (Gabel et al. 1977, p. 221). It seems that the growing concern about the drug’s toxicity in humans was changing the perspective of veterinarians, as evident when the *Equine Veterinary Journal* commissioned a review on clinical uses and side-effects by Jeffcott and Colles (1977) of the Animal Health Trust’s Equine Research Station. The day-to-day clinical practice of equine veterinarians, particularly in sporting contexts, is difficult to ascertain, in part because of the absence of records and in part, no doubt, due to secrecy in order to keep knowledge of treatment regimes away from competitors. Our best guides to practice and “experience”, then, come from reports like that written by Jeffcott and Colles.

There was a new factor too—animal welfare activists concerns with cruelty in horse racing. In May 1979, the Illinois Hooved Humane Society, an animal welfare organisation, issued its own report: “The misuse of drugs in horse racing: A survey of authoritative information on medication of racehorses”. This prompted a story in the *Chicago Herald* that warned: “A new drug culture is emerging in the United States—at the racetracks. More and more horses are getting by with a little help from their “friends”—butazolidin, lasix, and the illegal drugs some believe that they mask” (Korziol and Milbert 1979). New legislation was discussed in the US Senate and House of Representatives in “The Corrupt Horse Racing Practices Act, 1980”, which aimed to end “the drugging and numbing” of horses by instituting national legislative control of doping in racing (H.R. 7524 1980; see also S. 2636 1980; Bonnie 1982).^9^ For the legislators and advocates framing these bills, their main concern was the welfare of horses in training and running while injured, which then led to breakdowns and deaths in races (Tobin 1981). The bills failed in Congress and bute, along with Lasix, continued to be used “in competition” in US horseracing, albeit with different states allowing different dose levels. Veterinarians remained divided on banning the drug “out of competition”; with those opposed arguing that it allowed an injured horse to continue working to its long-term detriment. Meanwhile, in the UK, apparently stricter regulations were introduced in 1971, with phenylbutazone being banned “in competition” from all forms of horse racing (Lawrence 1971). However, given that the drug was known to clear the horse’s system relatively quickly, bute could be given to racehorses with veterinary approval and as long as administration ceased eight days before a race.

The fate of the drug in show jumping and eventing was more fraught, with disputes about its use “in competition”. In 1976, the horse Wide Awake collapsed and died in the show jumping phase at the Badminton Horse Trial. There was no obvious cause of death and suspicion fell upon bute, often given to horses on the third day of trials, to help them after the arduous second day cross-country round. Leading equestrians jumped to the defence of the drug, citing cross species differences, with Lucinda Prior-Palmer writing in the *Daily Telegraph*:

Some press coverage related the known effects of “bute” on humans to an effect which, it was imagined, might take place in horses. Not only has no such effect ever been recorded, but it is quite illogical to draw a scientific parallel between two species which are completely different (Prior-Palmer 1976, p. 56).

In 1980 the International Equine Federation (FEI) came under pressure to change its rules, which allowed phenylbutazone to be used “in” and “out of competition” (Macgregor-Morris 1976, 1980a). A ban was proposed by the Swedish delegation, but was overwhelmingly defeated, by 41 votes to 2. However, some members supported restrictions to prevent abuse and stricter monitoring was introduced (Smith 1980). By contrast, the FEI’s President, the Duke of Edinburgh, used his own cross-species experience to support the *status quo*: the drug was quite safe, he argued, as he had not only used it with his own polo ponies, but had taken it himself for a wrist injury (Anon 1980). Phenylbutazone administration and regulation then became a hot topic, with leading show jumpers threatening to leave the FEI if a ban was introduced. A compromise was agreed in 1980 and phenylbutazone was restricted to a maximum allowable blood level (Macgregor-Morris 1980b). Then, after the Duke’s daughter Princess Anne took over as President of the FEI, the allowable dosage was lowered from 4 μg ml^−1^ plasma to 2 μg ml^−1^ (Smith 1989). Bute was banned entirely in 1993, only to be allowed again in 2009, and after a year of continued controversy, using the drug “in competition” was finally banned again in 2010 (Green 1995; McArthur 2009; Report 2010).

While phenylbutazone’s use in equine sport dominated veterinary and public interest in the drug, the pressure to ban it in human medicine had prompted new enquiries into its use in horses. The *Equine Veterinary Journal* review in 1977 drew upon medical as well veterinary publications, but noted that the blood dyscrasias, which were of great concern in humans, had not been reported in horses (Jeffcott and Colles 1977). By contrast, veterinarians had only reported “minor” side-effects, such as water retention, depression, transient staggering, and phlebitis. The review’s conclusion was that, “despite the lack of documented evidence, the toxicity of phenylbutazone in the horse is lower than in man”, which they speculated was due to a combination of factors, including lower dose rates, faster physiological clearance, the type of condition treated and, of course, the species being treated. Later that year, the same journal published an editorial on bute in competition horses that began by reflecting on what its writers called “the objective world of science and the subjective rules governing social, economic and aesthetic attitudes of society” (Editorial 1981, p. 144). The unnamed editorialists went on to admit that veterinarians “knew far too little of the action of many therapeutic agents used in practice” and recognised “the wide variations between species in the pharmacokinetics of commonly used drugs”. The hope was that the investigation of such issues would not be driven by “the ethics of use of a drug in competitive sport”, but instead should “concentrate on furthering the knowledge of its action in our patients’” (p. 145).

The growth of veterinary work with non-sporting horses coincided with more reports on the adverse effects of bute in veterinary publications. In 1983, a report of toxicity in ponies described how high doses produced deaths and common side effects were swelling and ulceration of the mouth and gastrointestinal tract (MacAllister 1983). However, thoroughbred horses seemed to tolerate the drug much better, though veterinarians reported hypoproteinemia—lowered protein levels in the blood—in some horses (Snow et al. 1981). Also in 1983, Peter Lees and his colleagues at the Royal Veterinary College began studies that confirmed many previous findings (Lees et al. 1983a, b; Lees and Higgins 1985). They found evidence of kidney and liver damage, but concluded that such problems were manageable, with the best course of action being to avoid high dosages and withdraw the drug at any sign of side-effects. In 1986, a cross-Atlantic team, led by the University of Kentucky’s Thomas Tobin and the RVC’s Peter Lees, published an influential review of “Phenylbutazone in the Horse”. The review concluded that

In summary, it is now clear that early workers underestimated the toxicity potential of phenylbutazone. When given at high dose levels, even for short periods, accumulation, and hence toxic effects, can rapidly and readily occur. Toxicity appears as inappetence, melena, depression, mouth ulcers, diarrhea and possibly abdominal edema. If the drug is being administered in food, the condition tends to be self-limiting, since the animal will refuse to eat after a few days. If dosing is maintained, however, more serious toxicity and death may occur (Tobin et al. 1986, p. 21).

The review’s authors were confident that, with the recently adopted reductions in dosage and with proper hydration, “phenylbutazone should continue to be a safe and effective medication in the horse.” They based this recommendation on new clinical research findings, together with long clinical experience, which suggested, they argued, “that moderate doses can be given over prolonged periods without inducing clinically detectable side-effects” (Tobin et al. 1986, p. 21).

A quarter century later, in 2012, a similar review by the University of Pennsylvania’s Lawrence Soma reconsidered the issue of bute in equine sports (Soma 2012). His review focused mainly on the “moral dilemma” of allowing an injured horse to train and compete when “the medication contribut[ed] to further injury to the detriment of the horse”. The question of toxicity was again left to the side, as Soma noted that the “Blood dyscrasias commonly described in man have not been reported in the horse and despite the lack of documented evidence, toxicity of PBZ in the horse is considered to be lower than that in human” (Soma 2012, p. 2). Like the authors of the 1986 review, Soma relied upon veterinary experience and the absence of “documented evidence” on bute’s toxicity. However, there was a growing body of research literature that pointed to horses suffering some side-effects similar to those in humans: principally ulceration of the stomach and gut, kidney and liver damage (Higgins and Snyder 2006). The advice soon became to keep dosage low, avoid long-term use, monitor for toxicity, and consider alternatives, such as NSAIDs.

## Conclusion

At the height of the horsemeat scandal in 2013, Lees and Toutain (2013) wrote an editorial for *Equine Veterinary Education* aiming to demonstrate that “the illegal and erratic presence of trace amount residues of phenylbutazone in horse meat is simply not a public health issue” for humans (Editorial 2013, p. 273). Lees and Toutain reviewed the data on pharmacology, therapeutics, and toxicity in humans, horses and laboratory animals, noting marked species differences (See also Lees et al. 2004). With horses they were clear that the dosage regimes established in the 1980s resulted in few significant toxic effects, even when phenylbutazone was given over long periods (Lees and Toutain 2013). However, toxicity in humans was an entirely different matter, on which the authors detailed the well-known issues with the gastro-intestinal tract problems and blood dyscrasias, and also cited more recent concerns about carcinogenicity. Their key point was that adverse-effects in humans were dose-related and that the levels of phenylbutazone that humans were likely to receive from horsemeat were tiny. The accompanying editorial emphasised the point, quoting Professor Sir Colin Berry, Emeritus Professor of Pathology, Queen Mary, University of London, who had pointed out to consumers that they were “more likely to be hit by a meteorite than get aplastic anaemia from Bute via horse meat” (Editorial 2013, p. 274). In fact, the humans most at risk from bute these days are the athletes who take it illicitly during training, or the trainers and track workers who take veterinary bute for their own ailments (Carpenter and McDonnell 1995).

With hindsight it could be argued that the different fates of phenylbutazone in man, where it was banned, and horses, where it is still widely used, were determined by species biology. The drug is metabolised differently in the two species, being cleared twelve times quicker in horses than in humans. However, there were other differences that were equally, if not more, important. Phenylbutazone was used for different disease conditions, in different types of patient, with different dosages, and to achieve different outcomes. While the profile of why, when and how it was employed changed over time in both species, in humans phenylbutazone was prescribed mainly for chronic, arthritic diseases, while in horses it was for socially and economically, if not medically, acute conditions—to allow the “patient’s” participation in work, sport or recreation. In human medicine, the story of phenylbutazone is part of medical responses to the rise in the prevalence of chronic diseases, the development of steroid and non-steroidal anti-inflammatory drugs, and changing regimes of risk assessment, drug safety, and regulation. With horses, the focus was largely on equity, economics, and ethics in equine sports. Until the late 1970s and the moves to ban phenylbutazone in human medicine there were very few cross-species references, which is why, despite the drug’s widespread and high-profile use, doctors and veterinarians learned little from each other. However, from the 1980s onwards there were more cross-species translations and all one-way, from human to veterinary practice. This saw greater recognition of similar cross-species side-effects, but at frequencies that were hard to compare because of the different cultures of human and veterinary medicine, and the different status of their patients. Phenylbutazone remains a drug that is widely used in veterinary medicine, though because of cross-species influences, with ever greater caution. Indeed, perhaps more so since the horsemeat scandal, when the drug became a chemical and hence “objective” marker of what was an essentially cultural aversion to consuming horsemeat.

## Footnotes

1 See also Podolsky (2015, pp. 38).

2 Later the *Journal of Clinical Epidemiology*.

3 H.R. Frillmayer [memo], 17 March 1961, Box 3085, General Subject Files, Division of General Services, RG88 Records of the Food and Drug Administration, United States National Archives, College Park, Maryland (hereafter GSFiles/FDA Records).

4 F. Leslie Hart [memo], 12 April 1955, Box 1991, GSFiles/FDA Records.

5 H. W. Chadduck to Mrs. FHV, 19 March 1963, Box 3583, GSFiles/FDA Records.

6 H. W. Chadduck [memo], 31 March 1965, Box 3754, GSFiles/FDA Records.

7 Knowledge of these side-effects for specific groups of patients was not new: see for instance Leonard (1953).

8 In the early 1970 s, Geigy had merged with Ciba, another large Swiss pharmaceutical firm, to form Ciba-Geigy. In 1996, Ciba-Geigy merged with Sandoz to form Novartis.

9 The catalyst for these bills was one put forward in the spring of 1979 by the Humane Society of the United States entitled the Drug Free Racing Act. These measures were aimed to control the use of phenylbutazone and furosemide (Lasix), a diuretic agent.
